# Salient type 1 interleukin 1 receptor expression in peripheral non-immune cells

**DOI:** 10.1038/s41598-018-19248-7

**Published:** 2018-01-15

**Authors:** Anping Song, Ling Zhu, Gowthami Gorantla, Olimpia Berdysz, Stephanie A. Amici, Mireia Guerau-de-Arellano, Kathryn M. Madalena, Jessica K. Lerch, Xiaoyu Liu, Ning Quan

**Affiliations:** 1 Department of Oncolgy, Tongji Hospital, Huazhong University of Science and Technology Tongji Medical College, Wuhan, Hubei 430030 P. R. China; 2 Institute for Behavioral Medicine Research, College of Medicine, The Ohio State University, Columbus, OH 43210 USA; 3 West China School of Basic Medical Sciences and Forensic Medicine, Sichuan University, Chengdu, Sichuan 610041 P. R. China; 4 School of Health and Rehabilitation Sciences, Division of Medical Laboratory Science, College of Medicine, Wexner Medical Center, The Ohio State University, Columbus, OH 43210 USA; 5 Department of Microbial Infection and Immunity, The Ohio State University, Columbus, OH 43210 USA; 6 Department of Neuroscience, The Ohio State University, Columbus, OH 43210 USA; 7 Center for Brain and Spinal Cord Repair, The Ohio State University, Columbus, OH 43210 USA; 8 Division of Biosciences, College of Dentistry, The Ohio State University, Columbus, OH 43210 USA; 9 Institute for Behavioral Medicine Research, 460 Medical Center Drive, Columbus, OH 43210 USA

## Abstract

Interleukin 1 is a pleiotropic cytokine that mediates diverse functions through its receptor, type I interleukin 1 receptor (IL-1R1). Most previous studies have focused on the expression and function of IL-1R1 in immune cells. Here we performed a comprehensive mapping of IL-1R1 distribution in multiple peripheral tissues using our IL-1R1 reporter (IL-1R1^GR/GR^) mice. This method yielded the highest sensitivity of *in situ* detection of IL-1R1 mRNA and protein. Besides validating previously reported IL-1R1 expression in the endocrine tissues including pituitary and pancreas, our results refuted previously reported exclusive IL-1R1 expression in neurons of the spinal cord dorsal horn and dorsal root ganglia (DRG). Instead, IL-1R1 expression was detected in endothelial cells within DRG, spinal cord, pancreas, colon, muscles and many immune organs. In addition, gp38^+^ fibroblastic reticular cells (FRCs), rather than tissue macrophages or other immune cells, were found to express high levels of IL-1R1 in colon and many immune organs. A functional test of spleen FRCs showed that they responded rapidly to systemic IL-1β stimulation *in vivo*. Taken together, this study provides a rigorous re-examination of IL-1R1 expression in peripheral tissues and reveals tissue FRCs as a previously unappreciated novel high IL-1R1-expressing cell type in peripheral IL-1 signaling.

## Introduction

Interleukin 1 (IL-1) is a pro-inflammatory cytokine that mediates physiological and pathological responses to stress and infectious and sterile inflammatory stimuli. In the periphery, IL-1 is known to have three major functions: 1) IL-1 promotes inflammation, development and maturation of immune cells^1,2^; 2) IL-1 regulates insulin levels^3^, glucose disposal^4^ and lipid metabolism^5^; 3) IL-1 impacts neuroendocrine system through activation of hypothalamic-pituitary-adrenal (HPA) axis^6^.

Previous studies have shown that almost all IL-1 actions are mediated by the type I IL-1 receptor (IL-1R1). Due to the diverse functions of IL-1, efforts have been made to map the distribution of IL-1R1 in peripheral tissues. Thus far, IL-1R1 has been found in all cells of innate immune system including macrophages, neutrophils, eosinophils, basophils, mast cells, as well as selective T cell populations in the adaptive immune system^1,7,8^. There is a bias in the literature on the distribution of IL-1R1 expressing cells because although hundreds of reports have shown IL-1R1 expression in immune cells, the expression of IL-1R1 in peripheral non-immune cells is largely neglected. Emerging evidence suggests that IL-1 can directly act on many non-immune cell types. For example, IL-1β can stimulate synovial fibroblasts and lymph node fibroblastic reticular cells to upregulate VEGF^9,10^. IL-1β can also control chemokine secretion in intestinal stromal cells^11^ and induce major acute-phase reactant production in hepatocytes^12^. The evidence thus far supporting the expression of IL-1R1 on non-immune cells were generated mostly by *in situ* hybridization^12^, Northern blot^13^, mRNA qPCR analysis^12^ or indirectly by comparison of cell signaling between wild type and IL-1R1 deficient mice^11^. However, a clear demonstration of IL-1R1 expression at protein level by immunohistochemistry or at mRNA levels with precise identifications of cell types is still missing, potentially due to the low expression level of IL-1R1. Moreover, in some cell types such as muscle fibers, IL-1R1 expression level is undetectable despite of the presence of IL-1 signaling^14^. Therefore, a more sensitive detection method for IL-1R1 could help illustrate the major IL-1 signaling targets in the periphery.

We have previously developed an IL-1R1 reporter mouse model, namely the IL-1R1 globally restored mice (IL-1R1^GR/GR^), to study IL-1R1 expression in the brain^15^. This mouse model allows tracking of IL-1R1 mRNA expression by the knockin tdTomato fluorescence and the IL-1R1 protein expression by the HA tag under the control of the endogenous IL-1R1 promoter. In the present study, we performed immunohistochemical analysis of the IL-1R1^GR/GR^ mouse tissues to examine the pattern of IL-1R1 expression in multiple peripheral tissues. Surprisingly, the highest IL-1R1-expressing cells in colon, muscle, and many immune organs were identified as fibroblastic reticular cells and endothelial cells, rather than leukocytes. These cells also rapidly responded to *in vivo* IL-1β stimulation with upregulation of the immediate early gene c-*fos*. These results reveal structural non-immune cells, rather than the mobile immune cells, as the preeminent peripheral IL-1R1 expressors and suggest these cells are the first and most critical responders to systemic or local IL-1.

## Results

### Identification of IL-1R1 expressing cells in the endocrine system

**Figure 1:**
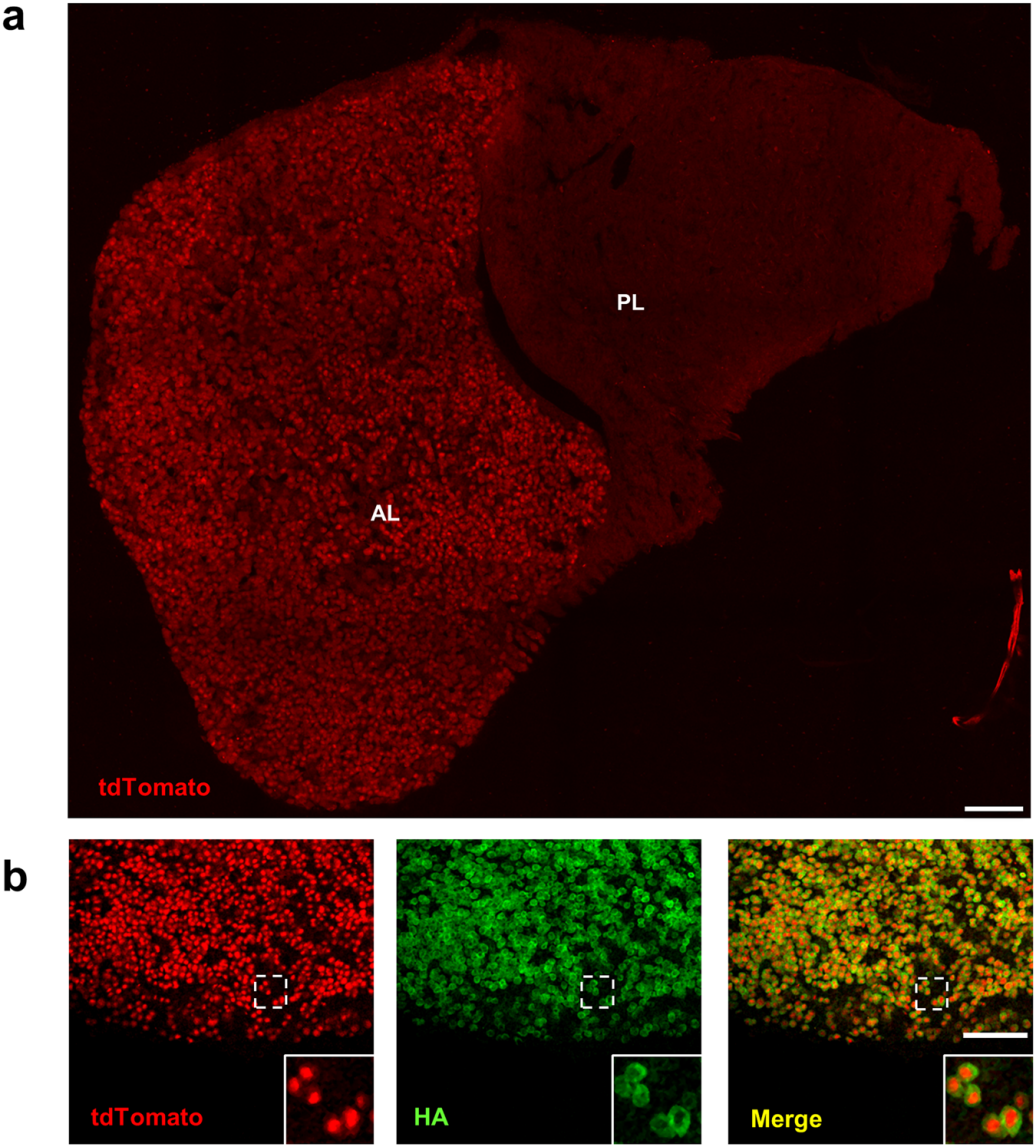
Pituitary IL-1R1-expression. (**a**) A representative collage image of pituitary sections from IL-1R1^GR/GR^ mice. (**b**) Immunofluorescence labeling of tdTomato and HA in the pituitary. Dashed square marks the area shown at higher magnification on the bottom right. AL, anterior lobe; PL, posterior lobe. Scale bar, 100 µm.

We have previously demonstrated that, in the IL-1R1^GR/GR^ mice, IL-1R1 mRNA-expressing cells are faithfully tracked by co-expressed tdTomato fluorescent protein^15^. Here we first investigated IL-1R1 expression in the endocrine system as IL-1 was known to activate the HPA-axis. Robust tdTomato fluorescence was detected in cells of the anterior, but not the posterior, lobe of the pituitary gland (Fig. 1a). Based on our genetic design, IL-1R1 protein in the IL-1R1^GR/GR^ mice is tagged by 3HA epitope^15^. IHC labeling of HA in the anterior lobe of pituitary showed that tdTomato^+^ cells colocalized with HA-labeled cells (Fig. 1b). At the subcellular level, the tdTomato labeling was surrounded by HA labeling, consistent with the fact that IL-1R1 mRNA stays in the cytoplasm whereas the IL-1R1 molecule is a membrane protein expressed on the cell surface (Fig. 1b insets).

**Figure 2:**
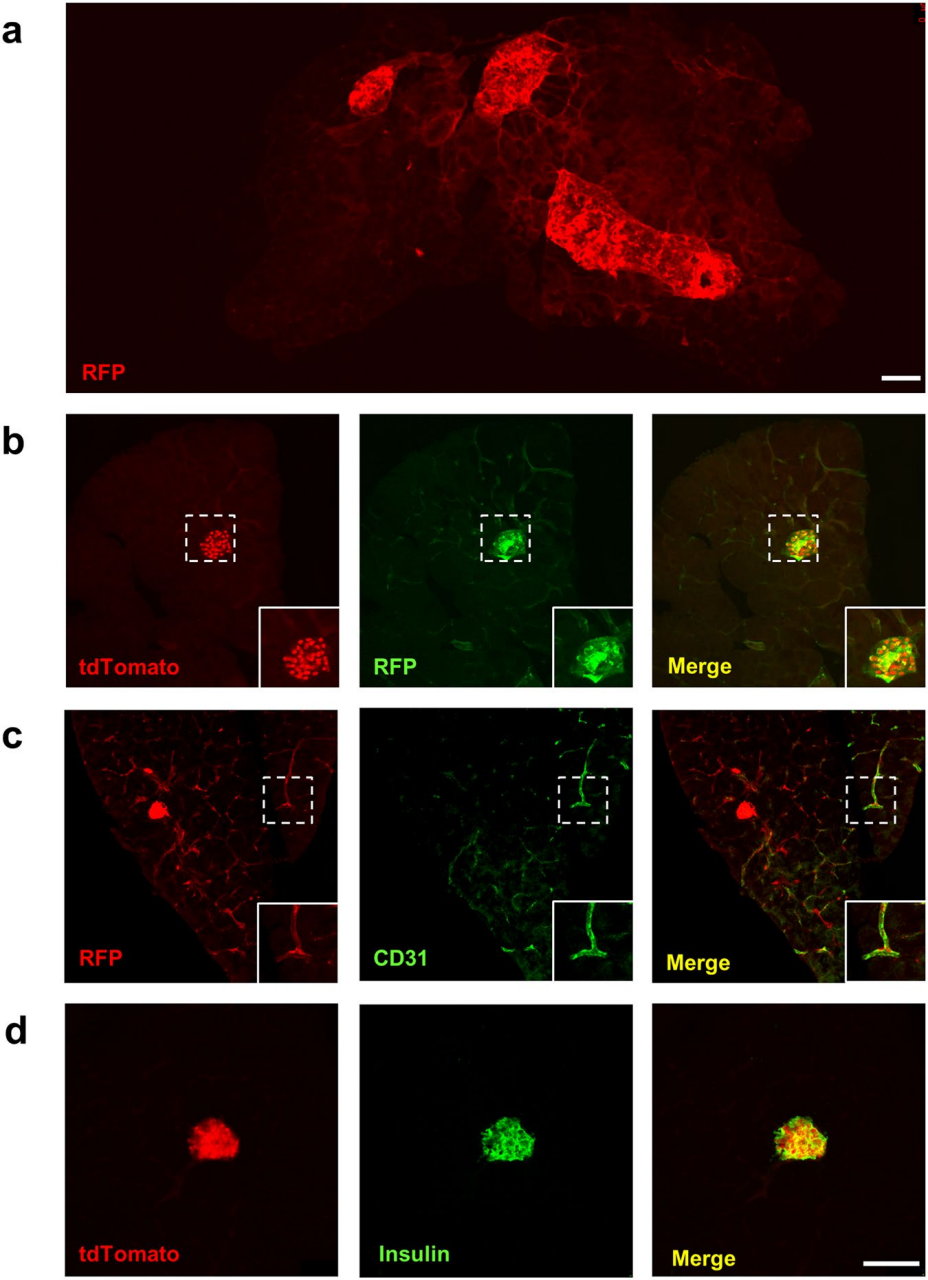
Identification of pancreatic IL-1R1-expressing cell types. (**a**) A representative collage of RFP fluorescence image of pancreatic sections from IL-1R1^GR/GR^ mice. (**b**–**d**) Immunofluorescence labeling of tdTomato (**b**,**d**), RFP (**b**), CD31 (**c**) and insulin (**d**) in the pancreas. Dashed square marks the area shown at higher magnification on the bottom right. Scale bar, 100 µm.

IL-1R1 expression has been reported previously in pancreatic β cells^16^. Hence, pancreas from IL-1R1^GR/GR^ mice was examined for IL-1R1 expression. To increase the detection sensitivity of IL-1R1 mRNA, we conducted IHC labeling of RFP (which detects tdTomato) to amplify the signal intensity of tdTomato protein. In Fig. 2b, IHC labeling of RFP colocalized with tdTomato^+^ cells with significantly stronger fluorescence signals, allowing more IL-1R1 mRNA-expressing cells to be identified. A collage of RFP labeling at low magnification showed robust IL-1R1 mRNA expression primarily in pancreatic islets and blood vessels (Fig. 2a). Double labeling of RFP or tdTomato with CD31 and insulin showed that the pancreatic IL-1R1 mRNA-expressing cells were endothelial cells and β cells (Fig. 2c,d). We also examined IL-1R1 expression in the adrenal glands but failed to detect any RFP labeling (data not shown).

### Identification of IL-1R1 expressing cells in the immune system

**Figure 3:**
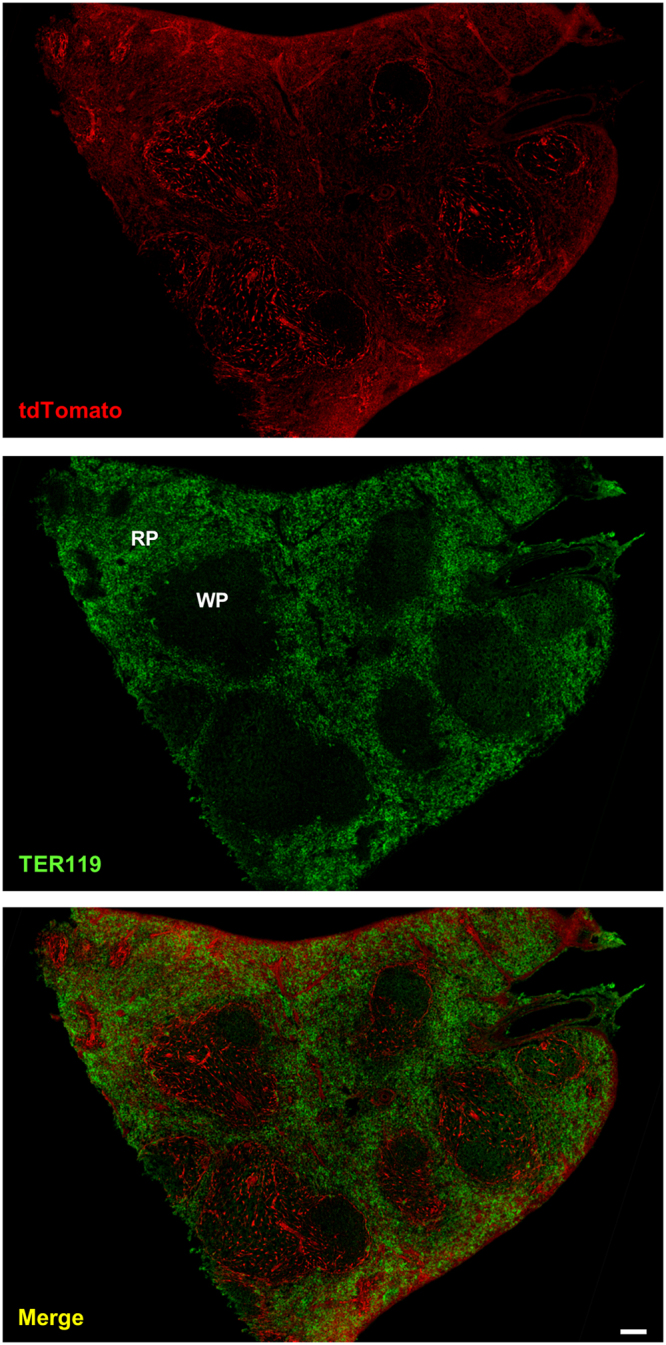
Representative collage images of spleen sections from IL-1R1^GR/GR^ mice with immunofluorescence labeling of tdTomato and TER119. RP, red pulp; WP, white pulp. Scale bar, 100 µm.

**Figure 4:**
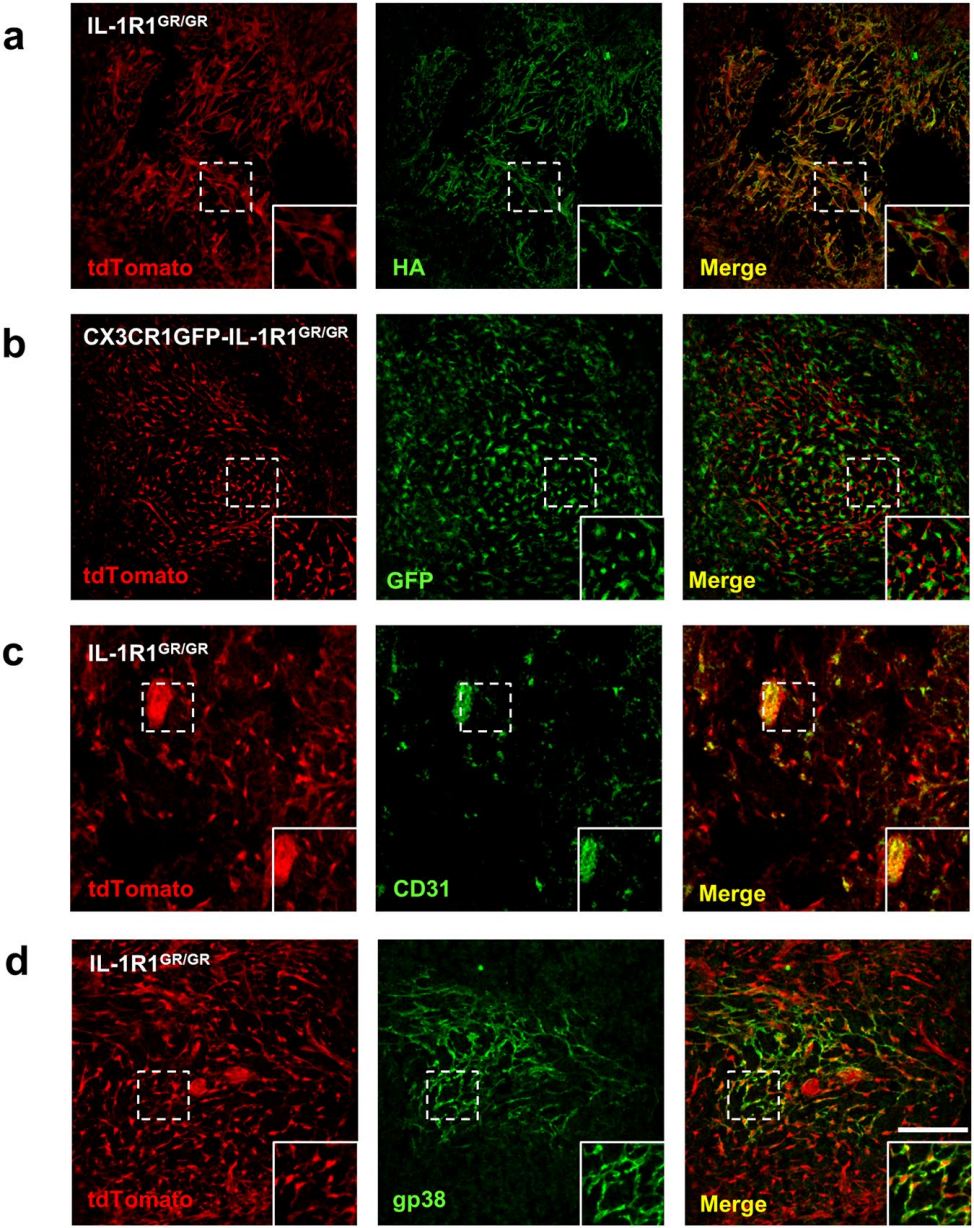
Identification of splenic IL-1R1-expressing cell types. (**a**–**d**) Immunofluorescence labeling of tdTomato (**a**–**d**), HA (**a**), CD31 (**c**) and gp38 (**d**) in spleen sections from IL-1R1^GR/GR^ mice, and GFP (**b**) in spleen sections from CX3CR1GFP-IL-1R1^GR/GR^ mice. Dashed square marks the area shown at higher magnification on the bottom right. Scale bar, 100 µm.

To identify IL-1R1-expressing cells in the immune system, we first examined spleen tissues. Strong tdTomato fluorescence, without RFP IHC amplification, was found in the spleen of the IL-1R1^GR/GR^ mice. Double labeling of tdTomato with erythrocyte marker TER-119 showed that tdTomato^+^ cells resided in the TER-119 negative white pulp of the spleen (Fig. 3). Immunostaining of HA was then done on the spleen sections. Similar to the results from the pituitary, immunofluorescence of HA was found strictly surrounding the tdTomato-labeled cell bodies, demonstrating again that IL-1R1 mRNA and IL-1R1 protein were expressed in the same cells (Fig. 4a insets). To identify the cell types of these IL-1R1-expressing cells, we examined whether they were spleen macrophages, which reportedly express functional IL-1R1^4,17^. To visualize spleen macrophages, IL-1R1^GR/GR^ mice were crossed with CX3CR1GFP knockin mice expressing enhanced GFP in monocytes, macrophages, dendritic cells (DCs) and natural killer cells (NKs)^18^. Surprisingly, tdTomato^+^ cells did not colocalize with GFP^+^ cells although the two cell types made physical contact (Fig. 4b insets), suggesting that the high IL-1R1-expressing cells in the spleen were not macrophages. Instead, they were identified as CD31^+^ endothelial cells and gp38^+^ fibroblastic reticular cells (FRCs), as shown in Fig. 4c and d. The immunohistochemical labeling pattern of gp38 delineated the cell membrane contours encircling the tdTomato-labeled cell body.

**Figure 5:**
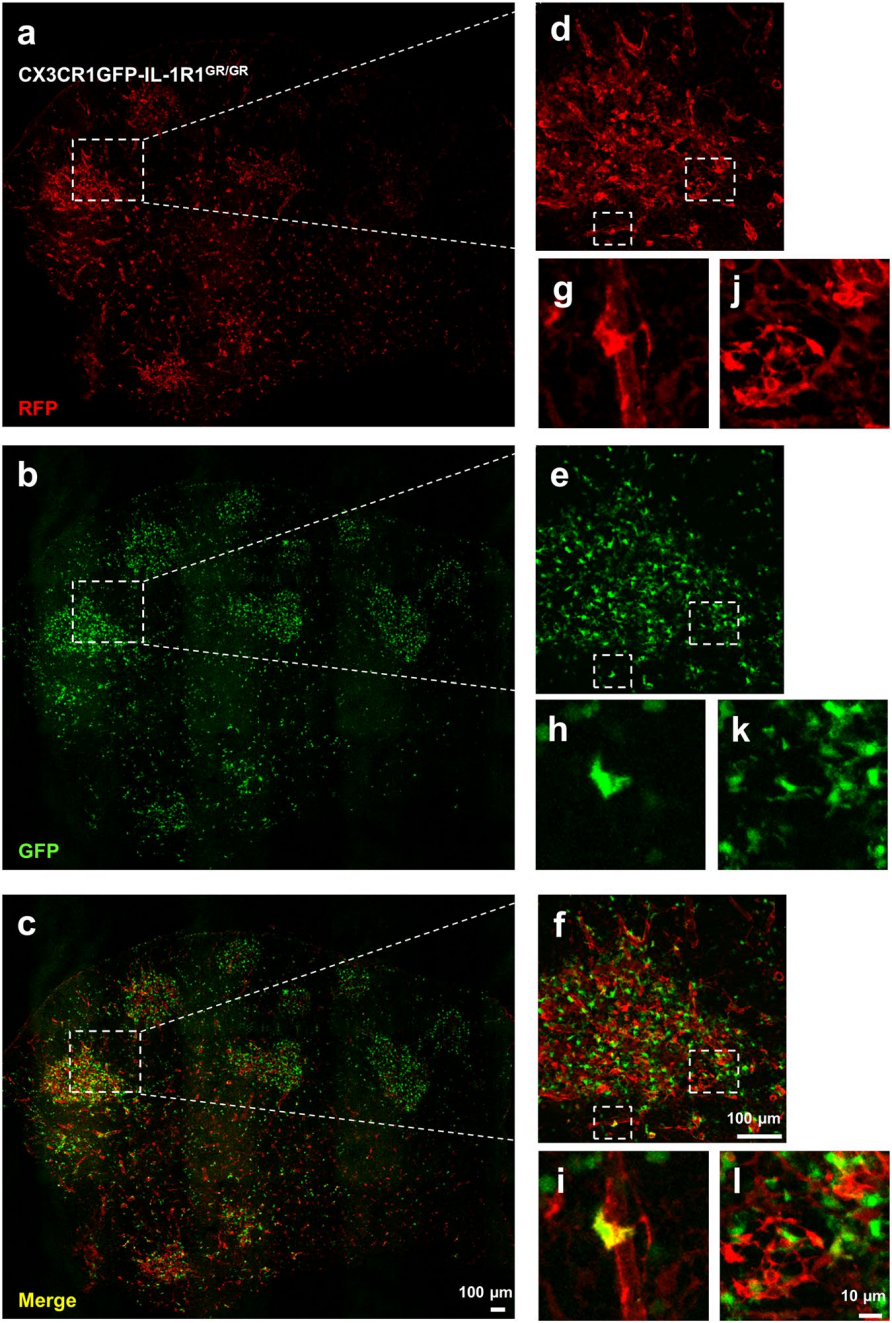
Immunofluorescence labeling of RFP and GFP in the thymus sections from CX3CR1GFP-IL-1R1GR/GR mice. (**a**–**c**) Representative collage images of thymus sections. (**d**–**f**) Higher magnification images of dashed square marked area in (**a**–**c**). (**g**–**l**) Higher magnification images of dashed square marked area in (**d**–**f**). Images (**g**–**i**) showed one RFP^+^ and GFP^+^ double labeled cells and image (**j**–**l**) showed few (less than 3) RFP^+^ and GFP^+^ double labeled cells in the corresponding areas. Scale bar for (**a**–**f**), 100 µm; for (**g**–**l**), 10 µm.

**Figure 6:**
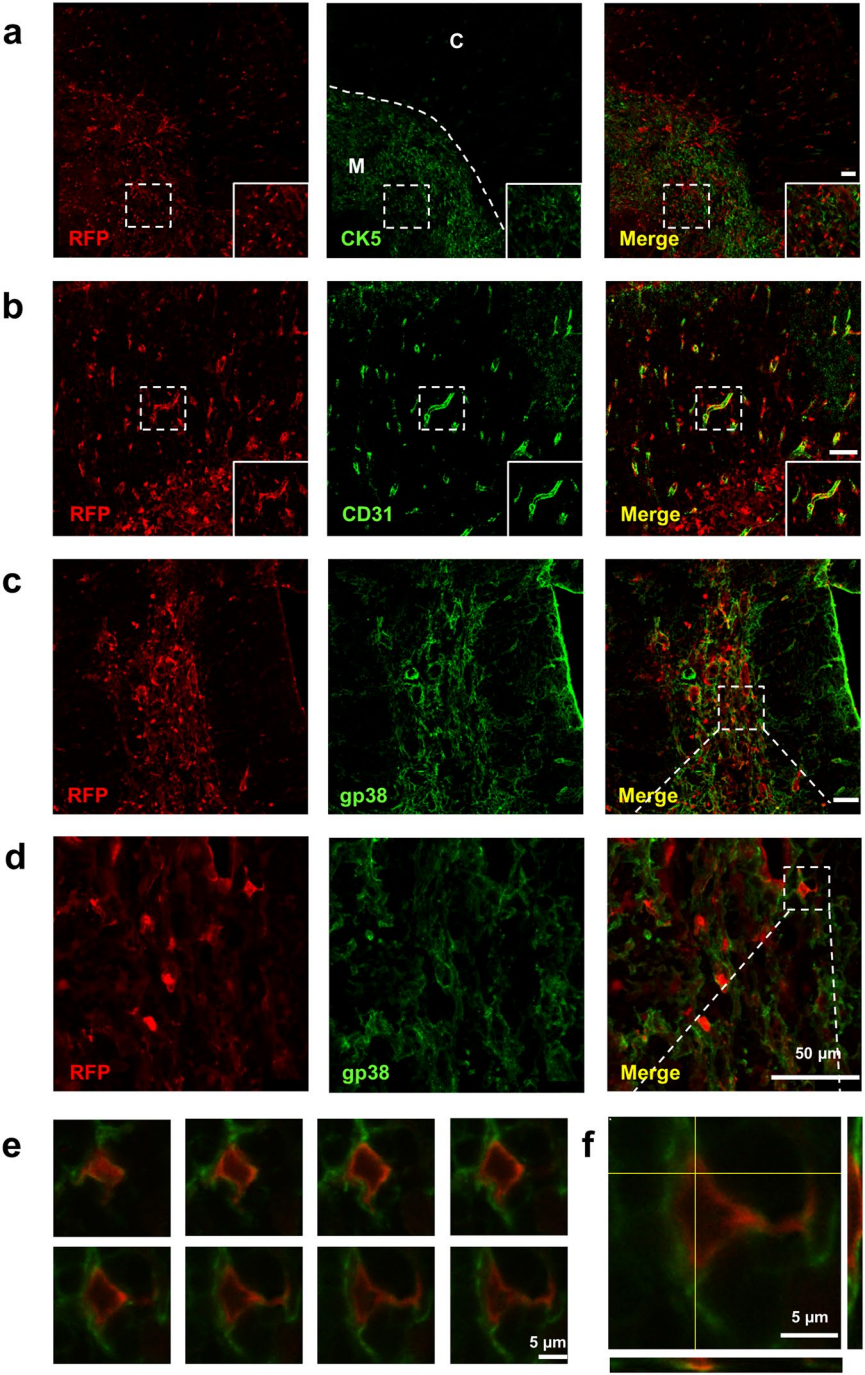
Identification of thymic IL-1R1-expressing cell types. (**a**–**c**) Immunofluorescence labeling of RFP (**a**–**c**), CK5 (**a**) CD31 (**b**) and gp38 (**c**) in thymus sections from IL-1R1^GR/GR^ mice. (**d**) Higher magnification images of dashed square marked area in (**c**). (**e**) Consecutive RFP and gp38 double labeled z-stack images at high magnification from dashed square marked area in (**d**). (**f**) Orthogonal view of one double labeled z-stack image (bottom left in (**e**)). Yellow lines represent vertical or horizontal optical cuts through the stack, which result in the side view images. M, medulla; C, cortex. Scale bar for (**a**–**d**), 50 µm; for (**e**–**f**), 5 µm.

Next, we investigated IL-1R1 expression in the thymus. In CX3CR1GFP-IL-1R1^GR/GR^ mice, RFP labeled IL-1R1 mRNA-expressing cells displayed different distribution patterns. Most RFP^+^ cells form clusters in selected regions where CX3CR1-expressing GFP^+^ cells also aggregated (Fig. 5). These regions were identified as the medulla of thymus by the thymic medullary epithelial cell marker Cytokeratin 5 (CK5). A closer examination of the RFP^+^ cells with double labeling showed that they were not thymic medullary epithelial cells (Fig. 6a). Instead, a few of the RFP^+^ cells were GFP^+^ CX3CR1-expressing cells or CD31^+^ endothelial cells (Fig. 5g,h,i and Fig. 6b). To identify the majority of IL-1R1 expressing cells, IHC labeling of gp38 were conducted (Fig. 6c–f). A series of consecutive double labeled images at high magnification from the z-stack images in Fig. 6d showed that fluorescent signals of RFP and gp38 targeted the same cells at different subcellular locations (RFP in the cytoplasm and gp38 on the cell membrane) at each focal plane (Fig. 6e). Furthermore, an orthogonal view of the images suggested that the two labeling signals merged at the boundary rather than one labeling being superimposed on the other (Fig. 6f). Thus, the thymic medullary IL-1R1-expressing cells were mostly FRCs with a few endothelial cells and CX3CR1^+^ macrophages/dendritic cells. Outside the medulla, sparse RFP labeled cells were found in the cortex of thymus. They were identified as CD31^+^ endothelial cells comprising the thymic blood vessels (Fig. 6b).

**Figure 7:**
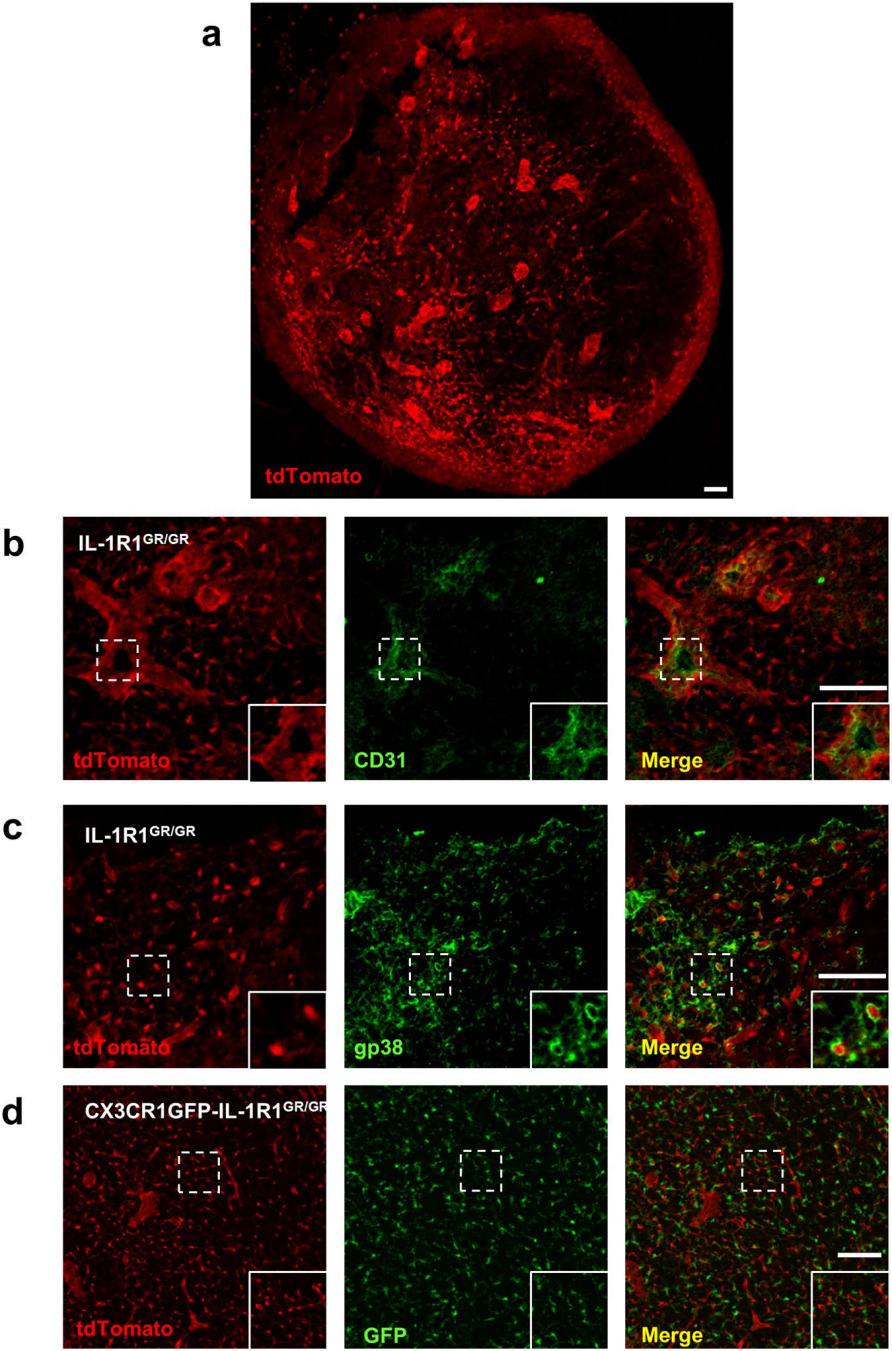
Identification of lymph node IL-1R1-expressing cell types. (**a**) A representative collage image of lymph node sections from IL-1R1^GR/GR^ mice. **(b**–**d**) Immunofluorescence labeling of tdTomato (**b**–**d**), CD31 (**b**) and gp38 (**c**) in the lymph node sections from IL-1R1^GR/GR^ mice, and GFP (**d**) in lymph node sections from CX3CR1GFP-IL-1R1^GR/GR^ mice. Dashed square marks the area shown at higher magnification on the bottom right. Scale bar, 50 µm.

Robust tdTomato fluorescence was detected in the lymph nodes of IL-1R1^GR/GR^ mice (Fig. 7a). Similar to results in the spleen, the high IL-1R1-expressing cells in the lymph nodes were found to be CD31^+^ endothelial cells and gp38^+^ FRCs rather than CX3CR1^+^ macrophages/monocytes (Fig. 7b–d). We also examined IL-1R1 expression in the bone marrow but did not detect any RFP labeling (data not shown).

### Identification of IL-1R1-expressing cells in the peripheral nervous system and other tissues

**Figure 8:**
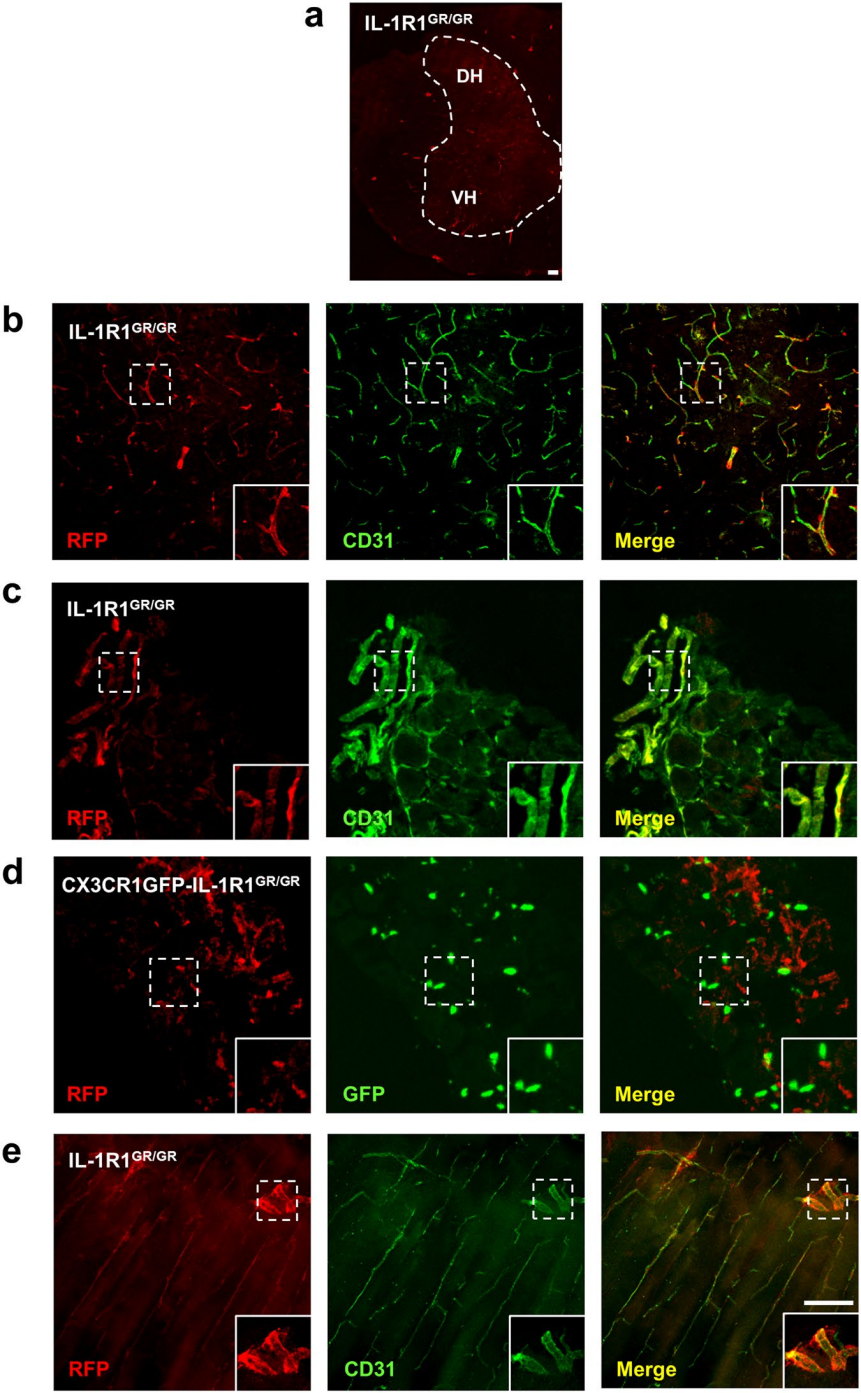
Identification of IL-1R1-expressing cell types in spinal cord, DRG and muscles. (**a**) A representative collage image of spinal cord sections from IL-1R1^GR/GR^ mice. (**b**,**c**) Immunofluorescence labeling of RFP and CD31 in the spinal cord sections (**b**) and DRG sections (**c**) from IL-1R1^GR/GR^ mice. (**d**) Immunofluorescence labeling of RFP and GFP in the DRG sections from CX3CR1GFP-IL-1R1^GR/GR^ mice. (**e**) Immunofluorescence labeling of RFP and CD31 in the muscle sections from IL-1R1^GR/GR^ mice. Dashed square marks the area shown at higher magnification on the bottom right. DH, dorsal horn; VH, ventral horn. Scale bar, 100 µm.

Previous studies have suggested that IL-1β induces mechanical and thermal allodynia through neuronal IL-1R1 in the spinal cord dorsal horn^19^ and dorsal root ganglia (DRG)^20–22^. Unexpectedly, IL-1R1 mRNA was not found in neurons or tissue macrophages in these regions in our IL-1R1^GR/GR^ and CX3CR1GFP-IL-1R1^GR/GR^ mice; this result was confirmed by NeuN, neurofilament and GFP immunolabeling (Fig. 8d and Supplemental Fig. 1). Instead, robust RFP fluorescence was found in the CD31^+^ endothelial cells in both DRG and spinal cord tissues, suggesting the high IL-1R1-expressing cells in the dorsal horn and DRG were endothelial cells (Fig. 8a–c).

**Figure 9:**
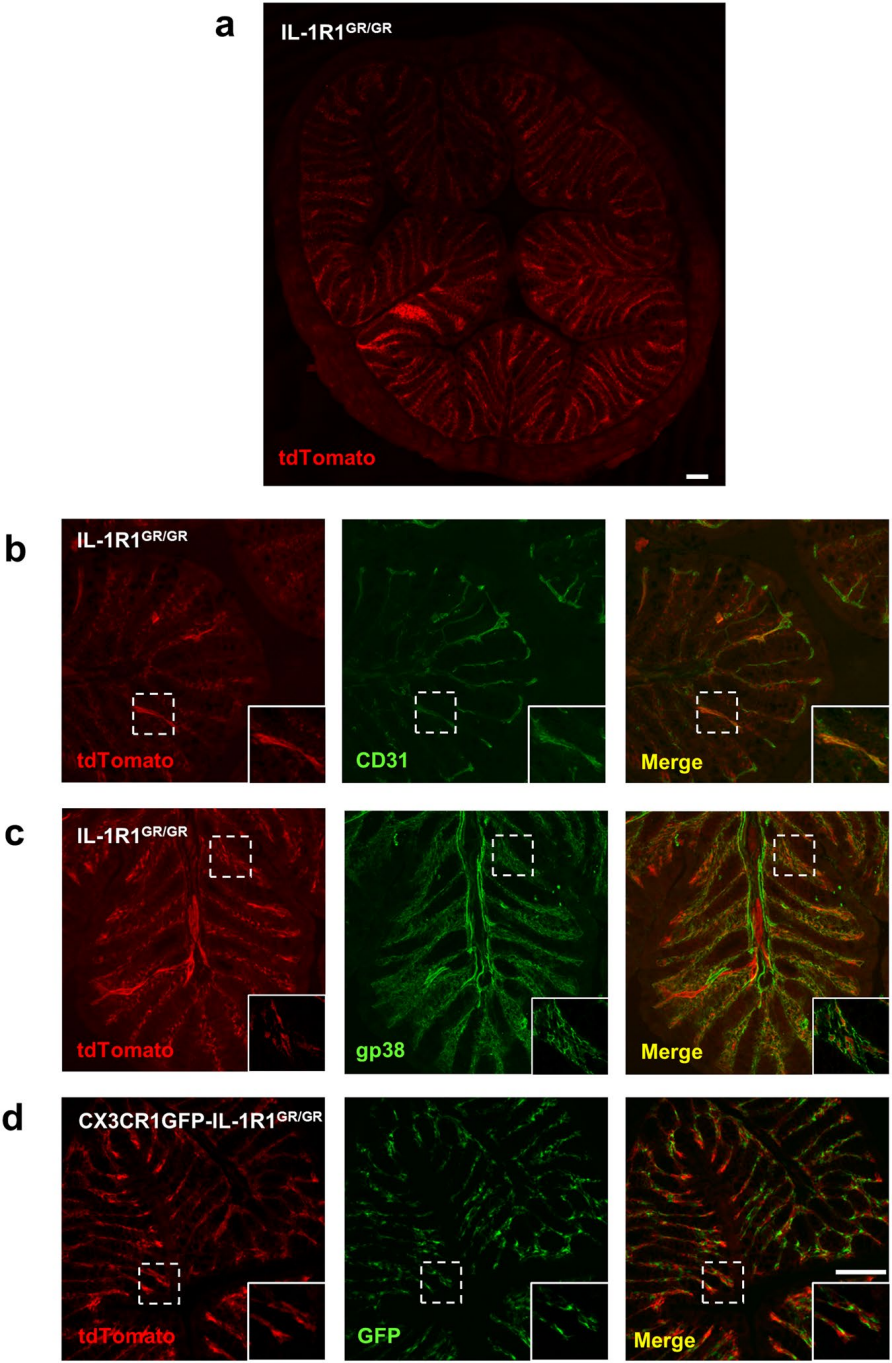
Identification of colonic IL-1R1-expressing cell types. (**a**) A representative collage image of colonic sections from IL-1R1^GR/GR^ mice. (**b**–**d**) Immunofluorescence labeling of tdTomato (**b**–**d**), CD31 (**b**) and gp38 (**c**) in the colonic sections from IL-1R1^GR/GR^ mice, and GFP (**d**) in colonic sections from CX3CR1GFP-IL-1R1^GR/GR^ mice. Dashed square marks the area shown at higher magnification on the bottom right. Scale bar, 100 µm.

We next investigated IL-1R1 expression in other tissues where IL-1 signaling was implicated. Similar to DRG, RFP labeling was detected only in the endothelial cells in the extensor digitorum longus muscles (Fig. 8e). In the colon, tdTomato labeled cells were observed in the lamina propria of the intestinal plicae. Double labeling of tdTomato with CD31 and gp38 showed that tdTomato^+^ cells were a few endothelial cells and mostly FRCs (Fig. 9b,c). In contrast, the GFP^+^ cells in the CX3CR1GFP-IL-1R1^GR/GR^ mice were found in close proximity to tdTomato^+^ cells without colocalization when examined at high magnification (Fig. 9d & insets), suggesting that the tdTomato^+^ cells were not colonic macrophages. Furthermore, no RFP signals were detected in the heart, kidney or liver although IL-1 mediated effects were reported in these tissues^23–25^.

### IL-1 induces c-*fos* expression in IL-1R1-expressing FRCs in the spleen

**Figure 10:**
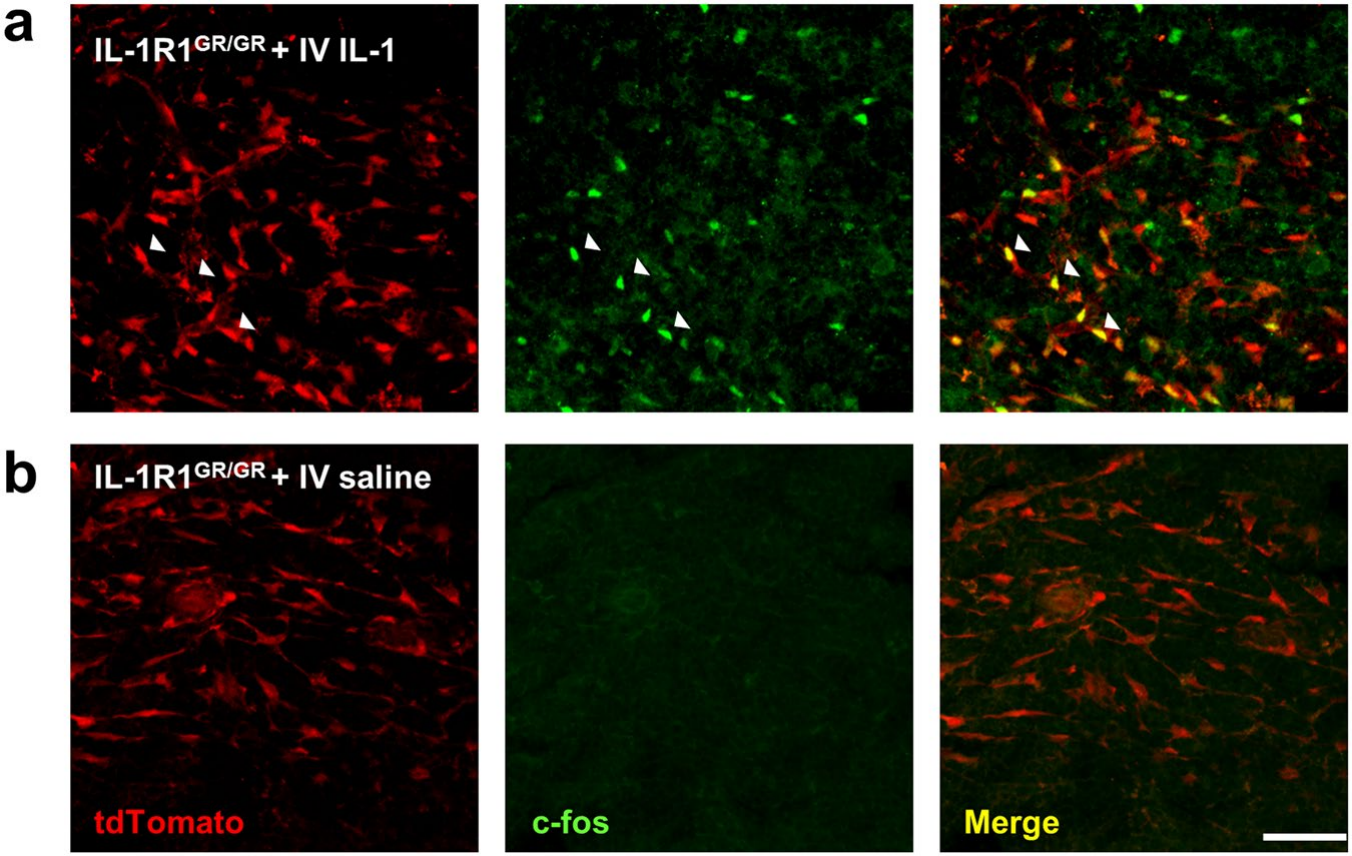
IL-1 induces c-fos expression in the IL-1R1-expressing FRCs in the spleen. Immunofluorescence labeling of RFP and c-*fos* in the spleen sections from IL-1R1^GR/GR^ mice 2 hrs after intravenous IL-1β or saline injections. Arrow heads showed RFP^+^ and c-*fos*^+^ double labeled cells. Scale bar, 50 µm.

Given that the majority of the high IL-1R1-expressing cells in the spleen were identified as FRCs, we next determined whether these cells could respond to *in vivo* IL-1β stimulation as a functional test of IL-1R1 mediated downstream signaling. Two hours after an intravenous injection of IL-1β, tdTomato^+^ c-*fos*^+^ double positive FRCs cells were localized in the white pulp of spleen (Fig. 10a). In contrast, no c-*fos* expression in the spleen was induced in the saline-injected control mice (Fig. 10b). Thus, IL-1β induced rapid transcriptional changes in the splenic IL-1R1-expressing FRCs.

## Discussion

In the present study, we performed a comprehensive and rigorous examination of IL-1R1 expression in multiple tissues using our IL-1R1 reporter (IL-1R1^GR/GR^) mice (Table 1). We confirmed the previously reported IL-1R1 expression patterns in the endocrine tissues including pituitary and pancreas with improved labeling at both mRNA and protein levels. We also discovered endothelial IL-1R1 expression in the spinal cord, pancreas, colon, DRG, muscles and many immune organs, which were not reported in the previous literature. In addition, we identified gp38^+^ fibroblastic reticular cells as the novel cell type expressing IL-1R1 at high levels in colon and multiple immune organs. A functional test of these cells showed that they responded rapidly to *in vivo* systemic IL-1β stimulation. This expression pattern of IL-1R1 reveals a novel IL-1 responsive structure in the periphery.

**Table 1:** Summary of IL-1R1 expressing cell types in multiple tissues examined in the current study.

Tissue	IL-1R1-expressing cell types
Pituitary	Hormone releasing cells in anterior lobe
Adrenal gland	Not detected
Pancreas	Beta cells, endothelial cells
Colon	Fibroblastic reticular cells, endothelial cells
Liver	Not detected
Spleen	Fibroblastic reticular cells, endothelial cells
Thymus	Endothelial cells, CX3CR1^+^ cells, fibroblastic reticular cells
Lymph nodes	Endothelial cells, fibroblastic reticular cells
Bone marrow	Not detected
Spinal cord	Endothelial cells
Dorsal root ganglia	Endothelial cells
Muscle	Endothelial cells
Heart	Not detected
Kidney	Not detected

Circulating IL-1 is known to promote metabolic changes through activation of HPA-axis and regulation of insulin production by pancreatic β cells^3,6^. Both pathways are mediated by IL-1R1 in the corresponding tissues. As a major component of the HPA-axis, the pituitary gland has been investigated for IL-1 signaling by many studies. Results of radio-ligand binding assays were the first to demonstrate that the anterior lobe of pituitary gland binds to radiolabeled IL-1α/β with high-affinity^26^. *In vitro* studies have also suggested that IL-1 stimulates the release of adrenocorticotropic hormone from both primary pituitary cell cultures^27^ and AtT-20 cells^28^, a mouse pituitary tumor line. Later, using *in situ* hybridization Cunningham *et al*. showed high levels of IL-1R1 mRNA in the anterior pituitary cells^29^. In the current study, we further improved the tracking of IL-1R1 mRNA by tdTomato fluorescence so that the morphology of the pituitary IL-1R1-expressing cells could be easily discerned. Additionally, the expression of IL-1R1 was confirmed at protein level by HA labeling. The congruent IL-1R1 protein and mRNA labeling in the present study attests the reliability of present results because potential artifacts due to single labeling of either the protein or mRNA can be excluded. We did not find any constitutive IL-1R1 labeling in the adrenal gland despite a few reports suggest the responsiveness of adrenal gland to acute IL-1β or LPS challenges^30,31^. It is possible that the adrenal IL-1R1 expression at physiological conditions is maintained at very low levels, but higher levels of IL-1R1 expression could be induced by immune challenges. This hypothesis can be tested using our IL-1R1^GR/GR^ mouse model in the future studies.

In the pancreas, IL-1R1 is known to be abundantly expressed in the insulin-producing pancreatic β cells^16^. Many early studies have shown that IL-1 has a potent inhibitory effect on insulin secretion, which results in islet destruction^32,33^. While studies indicating a deleterious role of IL-1 in the pancreas are largely based on the observation of islet dysfunction after high dose and chronic exposure to IL-1β, emerging evidence has suggested that IL-1 production at physiological levels contributes to many beneficial actions including stimulation of insulin secretion, glucose uptake^4^ and increase of lipase activity^5^. It remains unclear how IL-1 at different levels mediates opposite metabolic changes in the pancreas. Here we show IL-1R1 is expressed at a high level in the insulin-producing β cells, which is consistent with the previous reports, but at a much lower level in the pancreatic endothelial cells, which was not reported previously. As IL-1 signaling in the endothelial cells induces upregulation of cell adhesion molecules^34^, which promotes immune cell trafficking, endothelial IL-1R1 may account for the inflammatory effects of IL-1 in the pancreas. Therefore, our finding suggests that the pancreas could potentially respond to IL-1 in two different ways: the high IL-1R1-expressing islet β cells respond to low levels of circulating IL-1 with normal physiological actions such as increase of insulin production to maintain energy homeostasis, and the low IL-1R1-expressing endothelial cells react to high levels of IL-1 with local inflammatory actions, which ultimately lead to pancreatic islet destruction.

First discovered in circulating T cells^35^, IL-1R1 has been found to be expressed in all the innate immune cells and selective populations of the adaptive immune cells^1^. Myriad studies have demonstrated that IL-1R1 mediates the proliferation, expansion and maturation of the immune cells and their functions in inflammation and in antivirus and antitumor defenses. Surprisingly, here we show that the highest IL-1R1-expressing cells in the periphery are gp38^+^ fibroblastic reticular cells and endothelial cells rather than CX3CR1-expressing macrophages/monocytes or other immune cells. This expression pattern of IL-1R1 is detected in both immune and non-immune tissues including spleen, colon, thymus and lymph nodes, indicating a previously unappreciated role of these stromal cells in the modulation of inflammation. It should be noted that our IHC results in these tissues do not indicate complete absence of IL-1R1 expression in the immune cells. In fact, using the same mouse model, we have previously demonstrated that immune cells express functional IL-1R1, which is at a lower level than the detection limit of tdTomato fluorescence^15^. However, since traditional methods of immune cell isolation from peripheral tissues (without enzymatic digestion and dissociation) result in the loss of stromal cells, their functions in IL-1 signaling may have been largely missed. Additionally, the constitutive high level of IL-1R1 in the FRCs is functional, supported by the results that the FRCs rapidly (2 hrs) upregulate expression of an immediate early gene, c-*fos*, in response to *in vivo* IL-1 stimulation. In contrast, the low IL-1R1-expressing immune cells displayed a delayed cytokine response (24 hrs) to IL-1 stimulation, as shown by our previous report^15^. Based on the aforementioned data and the observation that FRCs locate near the immune cells, especially macrophage lineage cells (Figs 4b, 5, 7d and 9d), we speculate that: 1) circulating IL-1 stimulates FRCs before acting on the immune cells in most immune organs; 2) FRCs may play critical roles in collaborating with nearby macrophages to modulate local inflammatory activities. These interpretations can provide further insights into the understanding of how inflammation and infections influence immune cell development in both primary and secondary lymphoid organs. For example, it is known that T cell differentiation depends on the thymic microenvironment and the surrounding cytokine milieu^36^. Recent studies suggest that bacterial and viral infections inside the thymus can elicit thymic atrophy^37,38^, pathogen-specific T cell tolerance^39^ and alterations in T cell export^37^. As induction of pro-inflammatory mediators is a major hallmark of acute thymic infections and FRCs in the thymus is essential for development of medullar thymic epithelial cells^40^, it is possible that the aberrant T cell maturation is mediated by IL-1 signaling in thymic FRCs. In another study, IL-1 signaling is directly implicated in the inflammatory lymph node growth and dendritic cell activation^10^. Our findings on the FRC IL-1R1 expression may help explain the function of IL-1 in the vascular-stromal growth and suggest a novel pharmacological target in modulation of lymphocyte function in disease. Together, our results advocate a preeminent role of FRC in IL-1 mediated responses.

Previous reports found IL-1R1 expression in the spinal cord dorsal horn and DRG neurons, and assigned the neuronal IL-1R1 as the mediator of IL-1β-induced thermal and mechanical allodynia^19–22^. Another report showed IL-1 signaling in muscle fibers contributes to idiopathic inflammatory myopathies^14,41^. Here we show IL-1R1 expression only in endothelial cells but not in any other cell types in the muscles, spinal cord and DRG. It is possible that IL-1R1 levels on the neurons or muscle fibers are too low to be detected by our IL-1R1 reporter mice. In a more recent report using single-cell RNA sequencing to analyze DRG tissues, IL-1R1 mRNA is found to be at a markedly higher level in non-neuronal cells than in DRG neurons, which is in accordance with our results^42^. Another possibility for IL-1 mediated effects in these peripheral tissues is that IL-1 influences peripheral neurons or muscle fibers indirectly through IL-1R1 on endothelial cells, which then relay the signals to other cell types. This indirect mechanism may account for the results in *in vivo* models of arthritis pain and musculoskeletal pain inhibition of IL-1 signaling provides therapeutic efficacy^43,44^. Nevertheless, the present results do not support previous findings in which IL-1R1 was exclusively located to the neurons or muscle cells, but not endothelial cells.

Overall, the current study provides a thoroughly validated map of the IL-1R1 distribution in peripheral tissues. The discovery of novel IL-1R1-expressing cell types shows a major component of the IL-1 signaling system has been missed in the past.

## Methods

### Mice

The IL-1R1 globally restored IL-1R1^GR/GR^ mice and IL-1R1 global knockout IL-1R1^r/r^ mice were created by our lab previously^15^. In brief, the IL-1R1^GR/GR^ mice have normal IL-1R1 expression in all cell types under its endogenous promoter control. In addition, the GR allele contains 3HA and IRES-tdTomato sequence at the 3’ end of the IL-1R1 gene, allowing IL-1R1 mRNA expression to be tracked by tdTomato fluorescence and protein expression tracked by HA tag. IL-1R1^r/r^ mice were used as negative controls for the study. CX3CR1GFP-IL-1R1^GR/GR^ mice were generated by crossbreeding IL-1R1^GR/GR^ mice with CX3CR1GFP mice (stock #005582; Jackson Laboratories, Bar Harbor, ME). Male 6–8 week old mice were used in the current study. All mice were group housed in polypropylene cages, with food and water available *ad libitum*, in rooms maintained at 21 °C under a 12 h light/dark cycle. All procedures were in accordance with the National Institute of Health Guidelines for the Care and Use of Laboratory Animals and were approved by the Ohio State University Institutional Laboratory Animal Care and Use Committee.

### Immunohistochemistry

Mice were euthanized with carbon dioxide and perfused transcardially with cold 1 × PBS (pH 7.4), followed by 4% paraformaldehyde in phosphate buffer. After the perfusion, several tissues were dissected including spinal cord, pituitary, adrenal gland, femur, thymus, lymph nodes (inguinal, axillary and brachial), spleen, pancreas, colon, dorsal root ganglion and extensor digitorum longus muscles. These tissues were postfixed in 4% paraformaldehyde for 24 h, then equilibrated in a cryoprotective solution of 20% sucrose at 4 °C for 24 h and frozen at −80 °C. Mouse femurs were decalcified in 0.5 M EDTA in calcium and magnesium-free Dulbecco’s PBS (pH 7.8) at 4 °C for 24 h prior to freezing. Later, 30-µm-thick coronal sections were generated with a cryostat. Sections were placed free-floating in the cryoprotectant until staining. Sections were washed in PBS, blocked with 5% normal donkey serum (1% BSA, 0.1%TritonX in PBS), and incubated with primary antibodies. Primary antibody incubations were completed overnight at 4 °C. Sections were then washed in PBS and incubated with a fluorochrome-conjugated secondary antibody. Sections were mounted on slides and cover-slipped with Vectashield (Vector Laboratories). For tissues with high auto fluorescence background such as thymus, sections were treated with 0.7% Sudan black in 70% ethanol for 20 min before mounting.

For tissues difficult for free-floating immunostaining, including pituitary, lymph nodes and bone marrow, sections were generated using the CryoJane Frozen Sectioning Kit (Instrumedics Inc.; Hackensack, NJ) and on-slice immunostaining was conducted.

### Antibodies

The primary antibodies used for immunostaining were: anti-GP38 (1:33; goat anti-mouse, catalog #AF3244; R&D system), anti-HA (1:100; rabbit anti-mouse, catalog #3724; Cell Signaling), anti-RFP (1:1000; rabbit anti-mouse, catalog #Ab124754; Abcam), anti-CD31 (1:1000; rat anti-mouse, catalog #102502; Biolegend), anti-insulin (1:200; guinea pig anti-mouse, catalog #A056401; Agilent), anti-TER-119 (1:100; biotin rat anti-mouse, catalog #553672; BD Biosciences), anti-c-*fos* (1:1000; rabbit anti-mouse, catalog #sc-52; Santa Cruz), anti-NeuN (1:100, mouse anti-mouse, catalog #MAB377; Millipore Sigma). For immunostaining of NeuN, endogenous mouse IgG was blocked using the M.O.M kit (Vector Laboratories) prior to primary antibody incubation. For immunostaining of neurofilament, a three-antibody cocktail for three different molecular weights of neurofilament (NF) proteins (1:500; chicken anti-mouse NF; 68 kDa, catalog #NF-L; 160 kDa, catalog #NF-M; 200 kDa, catalog #NF-H; Aves Labs, Inc.) was used as the primary antibody.

The secondary antibodies (1:500; Thermofisher Scientific) used for immunostaining were: donkey anti-rabbit immunoglobulin G (IgG) polyclonal antibody conjugated with Alexa Fluor 488 (catalog #A-21206) or Alexa Fluor 594 (catalog #A-21209), donkey anti-rat IgG polyclonal antibody conjugated with Alexa Fluor 488 (catalog #A-21208), donkey anti-goat IgG polyclonal antibody conjugated with Alexa Fluor 488 (catalog #A-11055), donkey anti-mouse IgG polyclonal antibody conjugated with Alexa Fluor 488 (catalog #A-21202), goat anti-chicken IgG polyclonal antibody conjugated with Alexa Fluor 488 (catalog #A-11039), and streptavidin conjugated Alexa Fluor 488 (catalog #S32354). The secondary antibody used for immunostaining of insulin was donkey anti-guinea pig IgG polyclonal antibody conjugated with FITC (1:200; catalog #706-545-148; Jackson ImmunoResearch Laboratories Inc.).

### Intravenous (IV) IL-1β injection

IV IL-1β injection was conducted to identify IL-1β responsive cells in the spleen. Recombinant mouse IL-1β (R&D Systems, catalog #401-ML-005) was administered intravenously at a dose of 6 µg/kg body weight into IL-1R1^GR/GR^ mice and IL-1R1^r/r^ mice. Mice were perfusion fixed 2 hours after the injections. Spleen was dissected for IHC of c-*fos*.

### Immunofluorescence imaging and processing

IHC results were examined with a Leica TCS SP8 confocal microscope. Multiple-channel images were overlaid and each stack was z-projected using ImageJ. For IL-1R1 mRNA distribution mapping, series of confocal images were acquired by tile scans and stitched by Leica Application Suite X software to produce a collage of tissue image.

## Electronic supplementary material

Supplementary Information
