# Supplementary material for: Salient type 1 interleukin 1 receptor expression in peripheral non-immune cells
Source: Sci Rep. 2018 Jan 15;8:723. doi: 10.1038/s41598-018-19248-7 (PMC5768710; doi:10.1038/s41598-018-19248-7)
Supplement: Supplementary file 1 — Supplementary Information [file 41598_2018_19248_MOESM1_ESM.pdf]

Supplementary Information

Title

Salient type 1 interleukin 1 receptor expression in peripheral non-immune cells

Authors and Affiliations

Anping Song[1], Ling Zhu[2,3], Gowthami Gorantla[2], Olimpia Berdysz[2], Stephanie A. Amici[2,4], Mireia Guerau-de-Arellano[2,4,5,6], Kathryn M. Madalena[6,7], Jessica K. Lerch[6,7] Xiaoyu Liu[2,8,9], and Ning Quan[2,8,9]

[1] Department of Oncolgy, Tongji Hospital, Huazhong University of Science and Technology Tongji Medical College, Wuhan, Hubei, 430030, P.R.China

[2] Institute for Behavioral Medicine Research, College of Medicine, The Ohio State University, Columbus, OH 43210, USA

[3] West China School of Basic Medical Sciences and Forensic Medicine, Sichuan University, Chengdu, Sichuan, 610041, P.R.China

[4] School of Health and Rehabilitation Sciences, Division of Medical Laboratory Science, College of Medicine, Wexner Medical Center, The Ohio State University, Columbus, OH 43210, USA

[5] Department of Microbial Infection and Immunity, The Ohio State University, Columbus, OH 43210, USA

[6] Department of Neuroscience, The Ohio State University, Columbus, OH 43210, USA

[7] Center for Brain and Spinal Cord Repair, The Ohio State University, Columbus, OH 43210, USA

[8] Division of Biosciences, College of Dentistry, The Ohio State University, Columbus, OH 43210, USA

[9] Corresponding authors: Xiaoyu Liu and Ning Quan, Institute for Behavioral Medicine Research, 460 Medical Center Drive, Columbus, OH 43210, USA, liu.1933@osu.edu or quan.14@osu.edu

Figure S1

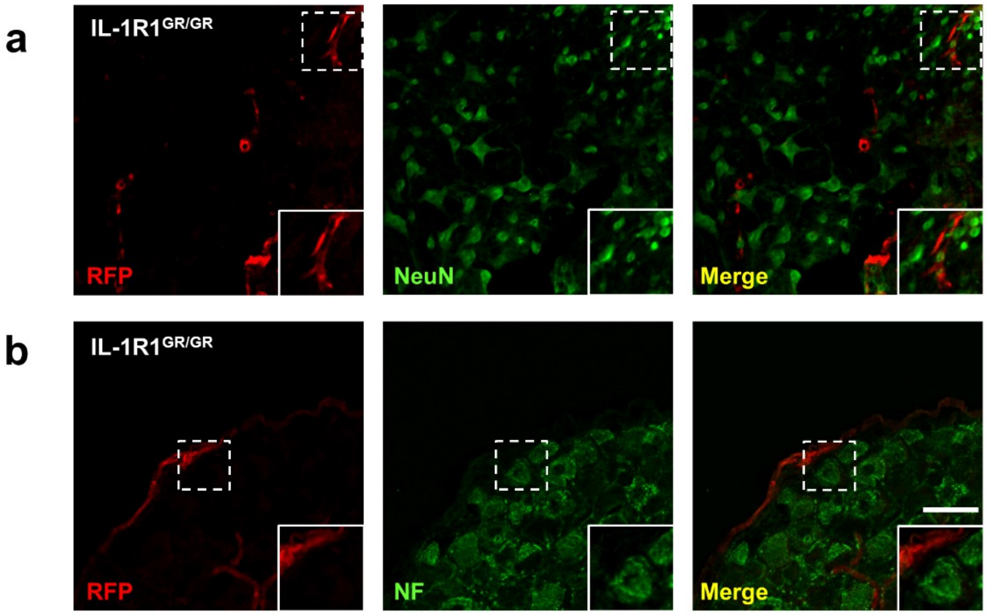

Supplemental Figure 1. Spinal cord and DRG neurons do not express IL-1R1. (a) Immunofluorescence labeling of RFP and NeuN in the spinal cord sections. (b) Immunofluorescence labeling of RFP and neurofilament (NF) in the DRG sections. Both sections were from IL-1R1$^{GR/GR}$ mice. Dashed square marks the area shown at higher magnification on the bottom right. Scale bar, 50 μm.
